# Associations of COVID-19 Risk Perception, eHealth Literacy, and Protective Behaviors Among Chinese College Students Following Vaccination: A Cross-Sectional Study

**DOI:** 10.3389/fpubh.2021.776829

**Published:** 2022-02-03

**Authors:** Ning Qin, Shuangjiao Shi, Guiyue Ma, Xiao Li, Yinglong Duan, Zhiying Shen, Aijing Luo, Zhuqing Zhong

**Affiliations:** ^1^Nursing Department, Third Xiangya Hospital, Central South University, Changsha, China; ^2^Xiangya School of Nursing, Central South University, Changsha, China; ^3^Department of Cardiology, The Third Xiangya Hospital of Central South University, Changsha, China; ^4^Department of Emergency, The Third Xiangya Hospital of Central South University, Changsha, China; ^5^Key Laboratory of Medical Information Research, College of Hunan Province, Central South University, Changsha, China

**Keywords:** COVID-19, vaccination, protective behaviors, risk perception, eHealth literacy

## Abstract

**Background:**

In spite of strict regulation of coronavirus disease 2019 (COVID-19) preventive measures and containment in China, there are still confirmed cases sporadically occurring in many cities. College students live in groups and have active social activities so that it will trigger a serious public health event once an infection event occurs. Thus, identifying the status and related factors of protective behaviors among them after receiving vaccination will be crucial for epidemic control. This study aimed to gather information on the protective behaviors and to identify the associations of COVID-19 risk perception, eHealth literacy, and protective behaviors for Chinese college students following vaccination.

**Methods:**

A cross-sectional survey of college students engaged in protective behaviors post vaccination was conducted using the COVID-19 risk perception scale, eHealth literacy scale, and protective behaviors following vaccination questionnaire in one of the groups. Multiple linear regression analysis was used to confirm the correlation among the COVID-19 risk perception, eHealth literacy, and protective behaviors for Chinese college students.

**Results:**

A total of 5,641 Chinese college students were included. Male students comprised 59.01% with an average age of (21.39 ± 2.75) years and most students rating their health as very good (44.85%) or pretty good (46.98%). A smaller percentage (13.76%) believed that they would likely or most likely be infected with COVID-19 after getting vaccinated. In addition, more than 1 in 10 (10.35%) college students had ever suspected to suffer from post-vaccination reactions following the COVID-19 vaccination. The mean score of protective behaviors was 26.06 ± 3.97. Approximately one-third (30.42%) of the students always or often did not wear a mask when going out. Some college students (29.25%) did not maintain distance of at least 1 m from others in social situations. Older female college students who were in good health and perceived as being at a low risk of getting infected with COVID-19, and those never suspected to suffer from post-vaccination reactions expected to engage in post-vaccination protective measures. Those with a higher level of perceived risk, severe risk perception and eHealth literacy, and a lower level of unknown risk perception were more likely to engage in further protective behaviors after getting vaccinated.

**Conclusions:**

Overall, the level of protective behaviors among the Chinese college students following vaccination could be improved, especially for male, younger college students in poor health. This study revealed the predictive effects of risk perception and eHealth literacy on protective behaviors, recommending that the negative and positive effects of risk perception should be balanced in epidemic risk management, and eHealth literacy promotion should also be emphasized for public health and social measures.

## Introduction

Coronavirus disease 2019 (COVID-19) is renowned for causing an infectious pneumonia which broke out at the end of year 2019 (1). Subsequently, the COVID-19 epidemic upgraded into a pandemic in January, 2020, and it was declared a public health emergency of international concern by the WHO. In March, 2020, the WHO had declared COVID-19 to be a pandemic. COVID-19 is characterized as a highly infectious and strongly pathogenic condition which posed serious threats to global health. As of June 25, 2021, there have been 179.69 million cases of COVID-19 diagnosed globally, of which about 3.90 million people have died (2). College students, a group of well-educated young people with a high Internet penetration rate, are characterized by active social contacts and intense cross-regional mobility. Additionally, college students live mainly in school groups, and have frequent contact with each other, which can easily lead to public health emergencies once a COVID-19 case is found. Thus, college students are the key group we should pay more attention for COVID-19 prevention and control and are among those who should be vaccinated. During the outbreak of COVID-19, college students showed good protective behaviors. Over two-thirds of American college students reported washing their hands at least six times a day (3) and most Sherubtse college students (93.5%) had good practice toward COVID-19 (4). Almost all Chinese college students were highly in favor of epidemic containment strategies and showed high adherence to them. Specifically, well over three quarters of college students performed well in frequent hand washing or hand hygiene (86.9%), in wearing face masks (92.8%), and in avoiding going out in public and hosting gatherings (91.2%) (5). The female students and those enrolled in post-graduate studies were inclined to take preventive measures (5); however, it is uncertain that how they will behave and respond to regular COVID-19 prevention and control at this phase after receiving COVID-19 vaccination, and the differences among individuals with varying sociodemographic characteristics require further analysis.

Risk perception is defined as the individual feelings and recognition to exterior objective risk which drive vital decisions on the behaviors (6). During the period of COVID-19, the risk perception for citizens was highly associated with their individual preventive measures against COVID-19. Savadori et al. found that the risk perception exerted positive effects on the preventive measures upon infection prevention for citizens (7). Specifically, those who felt anxiety toward COVID-19 were more inclined to engage in washing hands, wearing face masks, and maintaining social distance from others. Additionally, those who perceived themselves to be highly susceptible were inclined to reduce social contact (7). Alegria et al. identified positive correlation between frequency of washing hands with self-perceived effectiveness of washing hands and epidemic risk perception based on the Common-Sense Model of Self-Regulation (CSM-SR) (8). Chinese college students were associated with a relatively high risk perception toward COVID-19 in the study of Ding et al., especially in female students and non-medical students as 92.5% of them believed that those in good health condition may be infected with COVID-19, and 85.1% of the college students were concerned about members of their family becoming infected (9). Those with a higher level of risk perception were better informed with regards to COVID-19, as well as carrying out more preventive actions (9). The pandemic has been contained effectively as the research work continues on COVID-19, disease diagnosis and treatment go into standardization along with the regular prevention and control measures being placed into effect (10). At present, China has entered the phase of regular prevention and control, with the government offering free vaccines for COVID-19 for all of its citizens (11). The national vaccination plan has observed great progress due to the diligence of the government and its personnel combined with the active cooperation of Chinese citizens; however, sporadic cases have emerged in several regions in recent days (12). Under these circumstances, we sought to identify the risk perception of COVID-19 among the Chinese college students after receiving vaccination. In addition, we sought to confirm the association between the risk perception and preventive actions on infection prevention post vaccination.

During the time of COVID-19, home quarantine and limiting in person social contact were recommended and enforced globally. Public outings and social contact were confined on a large scale; thus, the internet and social media have become the main channels for citizens to grasp dynamic information and maintain social connections with one another. However, misinformation about COVID-19 shows up frequently on the internet. eHealth literacy plays a critical role in rapidly targeting high-quality information from the confusing online environment that is flooded with mixed messages, as well as in making correct decisions for practices. eHealth literacy is a fundamental skill from which individuals can benefit from eHealth services.

Norman et al. defined eHealth literacy as the ability to search, find, understand, and produce critical analyses of the targeted health information from online health resources, followed by making the correct decision to address health problems (13); it makes up an integral driving factor behind individual health behaviors (14, 15). Do et al. discovered that during the time of COVID-19, higher eHealth literacy was closely associated with the increased adherence to the actions on infection prevention and control (IPC) and maintenance of a better lifestyle (16). Nevertheless, frequent exposure to social media has its downside, which may lead to the reluctance of an individual to obtain or even refusing to obtain the COVID-19 vaccine. This may be the result of online spreading of false information about the COVID-19 vaccines (17). Still, as individuals with higher eHealth literacy perform better in critically evaluating online information, their decision-making is less likely to be influenced by misinformation. Li et al. have confirmed a positive moderating effect of eHealth literacy in social media use and preventative actions. Therefore, higher eHealth literacy is strongly correlated to an increased adherence to infection preventive actions (18). Li et al. found that Chinese college students showed a relatively high eHealth literacy during the time of COVID-19, and the higher eHealth literacy predicted better preventive behavior at a significant level (19). However, the eHealth literacy for Chinese college students under the phase of regular prevention and control remains to be seen. In addition, how eHealth literacy is associated with the preventive actions of individuals after receiving COVID-19 vaccines requires further investigation and analysis.

Based on the research questions, the present study sought to: gain knowledge of the preventive actions on infection defense, risk perception of COVID-19, and the eHealth literacy among Chinese college students, as well as discussing links between preventive actions with risk perception and eHealth literacy. This study presented not only as a basis for implementing measures of COVID-19 prevention and control among the Chinese college students, but also, as a reference for public health management on the COVID-19 prevention and control among Chinese college students from the perspective of risk perception and eHealth literacy.

## Materials and Methods

### Research Design and Recruitment of Participants

This cross-sectional study with onsite survey methods was conducted at a comprehensive university in Changsha city in the province of Hunan in China. The university, as one of key universities and colleges in China, offers 106-degree programs at the bachelor's level and enrolls more than 50,000 full-time students. The questionnaire QR code, downloaded from the online platform (Questionnaire Star, URL: https://www.wjx.cn/), was distributed as a paper questionnaire. College students were invited to scan the QR code on the campus from June 10 to 15, 2021. An informed consent form was initially signed online by all participants. Before participants filled in the questionnaire, the introduction, i.e., research background, purpose, rules of anonymity and confidentiality, and precautions would be explained to them. The inclusion criteria for participants consisted of: (a) college students over 18 years of age; (b) Chinese students from the target university; (c) completion of the COVID-19 vaccination; and (d) consent to participate in the survey. A total of 6,312 college students responded with 6,282 responses having met the inclusion criteria (1 person was under 18 years and 29 people who did not consent to this survey). We declared that the minimum time for completing the survey was 90 s, and 641 invalid responses were excluded for the time limit. In total, 5,641 valid responses were included in the data analysis (effective response rate was 89.37%).

### Study Measures

#### Demographic Information

Sociodemographic information included gender, age, major, education level, health condition, self-perception of susceptibility, and suspicion of suffering from post-vaccination reactions. Health condition status was based on self-report with response alternatives “Very good,” “Pretty good,” “in General level,” “Pretty poor,” and “Very poor.” Suspicion to suffer from post-vaccination reactions was measured using the question: “Have you ever suspected to suffer from post-vaccination reactions following the COVID-19 vaccination?” and responses were dichotomized as follows: “yes” and “no.”

#### COVID-19 Risk Perception

The COVID-19 risk perception was evaluated by the public health emergency risk perception scale (the PHERPS) compiled by Shen et al. in 2020 (20). The public health emergency in this survey was defined as COVID-19. The PHERPS included 9 items within 3 domains of dread risk perception (3 items), severe risk perception (3 items), and unknown risk perception (3 items). Each item was scored on a 5-point Likert scale from strongly disagree to strongly agree. The total score was the sum of 9 items with a range of 9–45. The higher the score, the higher the level of risk perception (Cronbach's α = 0.84).

#### eHealth Literacy

The eHealth Literacy scale (eHEALS) developed by Norman et al. in 2006 (21) was adopted by eHealth literacy. The Chinese version of eHEALS was developed from the English version and translated into Chinese by two graduate students who held an International English Language Testing System (IELTS) certificate. Afterward, the back-translation was performed by a professor who had previously studied abroad to ensure the accuracy of the translation. The eHEALS included 8 items with one domain. Each item was scored on a 5-point Likert scale from strongly disagree to strongly agree with a total score ranged from 8 to 40. The higher the score, the higher the level of eHealth literacy is (Cronbach's α = 0.96).

#### Protective Behaviors Following Vaccination

The questionnaire of protective behaviors was developed by the research team based on the COVID-19 advice for the public after getting vaccinated (22). It included 7 items within seven aspects, i.e., social distancing, mask-wearing, handwashing, sneeze protection, going-out limit, ventilating, and traveling limit. The score adopted a Likert 5 rating from never (1 point) to always (5 points). The item 2 was scored in reverse and the others were positive scores. The total score was the sum of the 7 items with a range of 7–35 (Cronbach's α = 0.73). The above instruments appeared in Multimedia Appendix 1.

### Data Analysis

For data analysis, IBM SPSS 25.0 (IBM Corporation, NY, USA) was used. For descriptive statistics, categorical variables were presented as *N* (%), such as gender, age groups, major, education level, health condition, self-perception of susceptibility, and suspicion of suffering from post-vaccination reactions. Continuous variables were presented as mean ± SD, such as age, the level of COVID-19 risk perception, e-health literacy, and protective behaviors after COVID-19 vaccination. A univariate analysis was performed using Student's *t*-test or one-way ANOVA. A multiple linear regression was employed to test the determinant factors affecting the protective behaviors. The multiple linear regression analysis with stepwise method (α_in_ = 0.05, α_out_ = 0.10) was conducted with the score of protective behaviors after vaccination as a dependent variable and the variables with statistical significance in univariate analysis, three domains of risk perception, and eHealth literacy as independent variables. Variable assignments were as follows. They were gender (1 = male; 2 = female), age (1 = 18 ~ 20 years; 2 = 21 ~ 23 years; 3 = ≥24 years), education (1 = undergraduate; 2 = post-graduate; 3 = PHD), health condition (1 = very good; 2 = pretty good; 3 = in General level, pretty poor, very poor), self-perception of susceptibility (1 = Impossible; 2 = Not likely; 3 = Likely, most likely), and suspicion to suffer from post-vaccination reactions (1 = yes; 2 = no). VIF ranged from 1.02 to 1.80, indicating that there was no multi-collinearity among selected independent variables. Statistical testing was bilateral with the statistical significance at *p* < 0.05.

## Results

### Socio-Demographic Characteristics

The results showed that among 5,641 investigated college students, male students accounted for 59.01% with the average age being (21.39 ± 2.75) years. The majority of this population were non-medical students (95.87%) and undergraduate students (73.11%), with 91.83% of them in very good or pretty good health condition. A small proportion (13.76%) thought they would likely or most likely be infected with COVID-19 after getting vaccinated. Besides, more than 1 in 10 (10.35%) college students had ever suspected to suffer from post-vaccination reactions after the COVID-19 vaccination (Table 1).

**Table 1:** Sociodemographic profiles and univariate analysis for protective behaviors following vaccination (*N* = 5,641).

**Variables**	***n* (%)**	**The score for protective behaviors** **(M ±SD)**	***t/F***	***P*-value**
**Gender**			−2.52	0.012
Male	3,329 (59.01)	25.95 ± 4.07		
Female	2,312 (40.99)	26.21 ± 3.83		
**Age (year)**			16.48	<0.001
18–20	2,507 (44.44)	25.86 ± 3.91		
21–23	2,071 (36.71)	25.98 ± 3.96		
≥24	1,063 (18.84)	26.68 ± 4.09		
**Major**			1.26	0.207
Medical	233 (4.13)	26.38 ± 3.91		
Non-medical	5,408 (95.87)	26.04 ± 3.98		
**Education**			12.65	<0.001
Undergraduate	4,124 (73.11)	25.90 ± 3.93		
Post-graduate	1,220 (21.63)	26.44 ± 4.12		
PHD	297 (5.27)	26.68 ± 3.82		
**Health condition**			80.61	<0.001
Very good	2,530 (44.85)	26.73 ± 4.19		
Pretty good	2,650 (46.98)	25.66 ± 3.68		
in General level, pretty poor, very poor	461 (8.17)	24.64 ± 3.70		
**Self-perception of the possibility of being infected with COVID-19 after vaccination**			13.50	<0.001
Impossible	838 (14.86)	26.69 ± 4.44		
Not likely	4,027 (71.39)	25.98 ± 3.87		
Likely, most likely	776 (13.76)	25.76 ± 3.88		
**Suspicion to suffer from post-vaccination reactions after vaccination**			−3.545	<0.001
Yes	584 (10.35)	25.51 ± 3.96		
No	5,057 (89.65)	26.12 ± 3.97		

*t, Two-sample t-test; F, one-way analysis of variance*.

### Characteristics of COVID-19 Risk Perception, eHealth Literacy, and Protective Behaviors

The mean score for COVID-19 risk perception was 36.82 ± 5.43. The domains of COVID-19 risk perception with declining mean scores were dread risk perception (12.93 ± 1.96), severe risk perception (12.93 ± 2.19), and unknown risk perception (10.96 ± 2.61). The mean score for eHealth literacy was 30.68 ± 7.16. Additionally, the mean score of protective behaviors for Chinese college students was 26.06 ± 3.97. The majority (60.96%) of college students always or often avoided going out into a crowd. Over two-thirds (70.74%) always or often canceled non-work or work-related trips. Some college students performed insufficient protective behaviors. Nearly one-third (30.42%) of the students always or often failed to wear a mask while going out. A fair portion of college students (29.25%) failed to maintain at least 1 m of distance from others in social settings (Table 2).

**Table 2:** The status of protective behaviors for Chinese college students following vaccination (*N* = 5,641).

**Protective behaviors**	**M ±SD**	**Frequency [*****n*** **(%)]**				
		**Never**	**Seldom**	**Sometimes**	**Often**	**Always**
Total score	26.06 ± 3.97	–	–	–	–	–
Keep at least 1 m away from others in social situations	3.08 ± 1.00	249 (4.41)	1,401 (24.84)	2,102 (37.26)	1,431 (25.37)	458 (8.12)
Do not wear a mask when going out	3.06 ± 1.03	468 (8.30)	1,457 (25.83)	2,000 (35.45)	1,403 (24.87)	313 (5.55)
Wash hands promptly after returning to your residence	3.80 ± 0.96	50 (0.89)	531 (9.41)	1,391 (24.66)	2,203 (39.05)	1,466 (25.99)
Cover any cough or sneeze in your bent elbow	4.30 ± 0.83	21 (0.37)	182 (3.23)	692 (12.27)	1,956 (34.67)	2,790 (49.46)
Avoid going to the crowded	3.74 ± 0.90	44 (0.78)	384 (6.81)	1,774 (31.45)	2,230 (39.53)	1,209 (21.43)
Open windows for ventilation to maintain air circulation	4.12 ± 0.77	13 (0.23)	114 (2.02)	925 (16.40)	2,696 (47.79)	1,893 (33.56)
Cancel non-work or work-related trips	3.95 ± 0.92	53 (0.94)	308 (5.46)	1,290 (22.87)	2,189 (38.81)	1,801 (31.93)

*M, Mean; SD, standard deviation*.

### The Factors Which Influence COVID-19 Protective Behaviors

As shown in Table 1, the univariate analysis indicated that the female college students, those above 24 years of age, those with a higher education level, those in superior health, those with a lower self-perception of susceptibility, and those never suspected to suffer from post-vaccination reactions were more likely to perform better in the personal protection (*p* < 0.05).

Results (Table 3) showed that nine determinant factors were reserved, i.e., gender, age, health condition, self-perception of susceptibility, suspicion of suffering from post-vaccination reactions, dread risk perception, severe risk perception, unknown risk perception, and eHealth Literacy, accounting for 14.2% of variation in predicting the level of protective behaviors.

**Table 3:** Multivariate linear regression analysis on the protective behaviors following the vaccination for Chinese college students (*N* = 5,641).

**Variables**	**Partial regression coefficient**	**Standard error**	**Standardized partial regression coefficient**	***t***	***P*-value**	***95% CI***
Constant	16.124	0.593	–	27.204	<0.001	(14.962,17.286)
Gender	0.528	0.102	0.065	5.196	<0.001	(0.329, 0.727)
Age (year)	0.370	0.066	0.070	5.626	<0.001	(0.241, 0.499)
Health condition	−0.750	0.081	−0.119	−9.246	<0.001	(−0.910, −0.591)
Self-perception of susceptibility to COVID-19	−0.190	0.095	−0.026	−2.000	0.046	(−0.376, −0.004)
Suspicion to suffer from post-vaccination reactions after the COVID-19 vaccination,	0.326	0.162	0.025	2.007	0.045	(0.008, 0.645)
Dread risk perception	0.381	0.032	0.188	11.917	<0.001	(0.319, 0.444)
Severe risk perception	0.099	0.030	0.055	3.289	0.001	(0.040, 0.158)
Unknown risk perception	−0.047	0.021	−0.031	−2.217	0.027	(−0.089, −0.005)
eHealth Literacy	0.125	0.007	0.225	17.874	<0.001	(0.111, 0.139)

*R^2^ = 0.142, F = 103.586, p < 0.001. CI, confidence interval*.

## Discussion

The results showed that the protective behaviors after COVID-19 vaccination for Chinese college students were positive in general and the majority of college students maintained good protective habits. Dread risk perception, severe risk perception, and eHealth literacy would positively predict the protective behaviors, but unknown risk perception had a negative predictive effect. Female college students, those who were older, in good health condition, with a lower self-perception of susceptibility, and those never suspected to suffer from post-vaccination reactions were inclined to have a higher level of protective behaviors. Under the circumstance of sporadic diagnoses which occurred in many cities, the results indicated a sufficient level of protection awareness among the Chinese students post vaccination. The COVID-19 risk perception was strong, which was closely correlated with the enforcement of strict measures to control the epidemic. Overall, our findings emphasized the importance of risk perception and eHealth literacy, and prepare policymakers and health managers to develop the necessary prevention policies and target education measures.

### Demographics and Protective Behaviors Following Vaccination

The results of this study suggested that female college students, those who were older, in good health condition, with less self-perception of susceptibility, those never suspected to suffer from post-vaccination reactions were more likely to perform better in the COVID-19 protection. This was confirmed *via* several former studies. Ferdous et al. (23) and Li et al. (24) found that the female, elder residents in good health condition will take precautions more frequently, which may be attributed to a higher level of cognition, a more precautious attitude toward COVID-19 as well as better compliance of the IPC guidance for those residents (25, 26). A previous study (23) in Bangladesh showed that those residents with a high level of education would perform better in the protective behaviors, and education showed a statistically significant correlation with the level of protective behaviors in this study during the univariate analysis. This was similar to the results of Olaimat et al., i.e., undergraduates performed worse in protective behaviors compared with graduate students in Jordan (27). However, it was insignificant in the multivariate linear regression. This may be related to the characteristics of education among college students, i.e., with more advanced age, higher levels of education were more prevalent and the education factor was adjusted in the regression analysis. A small proportion of those who were likely or most likely to infect with COVID-19 after being vaccinated or suspected to suffer from post-vaccination reactions performed significantly worse in protective behaviors, which may be due to their negative attitudes toward COVID-19 as well as lack of trust in the efficacy of vaccine for these college students (28). Wang et al. pointed out that lower self-perception of susceptibility and good protective behaviors were two important protective factors for good mental health during the COVID-19 outbreak (29). Mo et al. found that much greater concerns and self-perceived susceptibility toward COVID-19 were the risk factors for anxiety and depression (30). This suggested that more attention should be given in assessing the psychological state to prevent the rising of psychological health issues.

### COVID-19 Risk Perception and Protective Behaviors Following Vaccination

The results of this study showed relatively higher levels of dread risk perception and severe risk perception than that of unknown risk perception. Unknown risk perception was primarily associated with the level of cognition with regards to COVID-19 and the accuracy of detection and diagnosis (20), for which the results were determined by the development of COVID-19. During the pandemic, as a result of the low level of cognition and the lack of effective detection and diagnosis methods, the level of unknown risk perception for the college students was quite high. With the establishment of the global COVID-19 IPC guidance, the epidemic was gradually brought under control, the vaccination strategy was carried on methodically, and the level of unknown risk perception for the college students was improved side-by-side. In addition, the results suggested that different domains of risk perception had various effects on protective behaviors, i.e., the dread risk perception and severe risk perception had positive effects in contrast to unknown risk perception which played a negative role. This appeared to be inconsistent with previous studies. In the perspective of dread to evaluate the risk perception, Taghrir et al. found a negative relationship between protective behaviors and risk perception for Iranian medical students (31). Xu et al. found that the dread risk perception had a positive effect on COVID-19 protective behaviors among Chinese adults (32). However, Quandt et al. revealed that the actual protective behaviors may be poor when the self-efficacy level was low even if high levels of dread risk perception of COVID-19 was identified among the Latinx Farmworker and Non-farmworker Families in North Carolina (33). Jahangiry et al. indicated that either dread control (protective behaviors) or risk control (non-protective behaviors) may be adopted when the level of risk perception was high among the Iranian general population (34). This may be closely associated with the attitude toward COVID-19, the level of self-efficacy, and response efficacy. Xie et al. found the mediating effect of safety climate between risk perception and social distance among Chinese residents (35). Thus, there was not a simple linear relationship between risk perception and health behaviors, and multiple factors (i.e., sociodemographics, psychological factors, self-efficacy, and safety climate) exerted varying effects on the association routes between them. The findings of this study suggested that the relationship between risk perception and protective behavior should be considered to balance for COVID-19 risk management, i.e., mobilizing positive factors to promote protective behaviors (36) and avoiding excessive risk perception may deter from the negative effects, such as poor mental health (37).

### eHealth Literacy and Protective Behaviors Following Vaccination

Compared with health literacy, eHealth literacy may play a crucial role during the COVID-19 outbreak (38). Do et al. compared the effects of health literacy and eHealth literacy on the compliance with COVID-19 infection control measures of healthcare workers and found that the latter had a more positive effect (16). The results of this study showed that eHealth literacy positively predicted the protective behaviors of college students post vaccination. This corresponded to previous findings (39) and emphasized the importance of promoting eHealth literacy and the necessity of the related educational programs in epidemic prevention and control. However, the positive effects seemed to be weaker in this study (β = 0.125) than those in the COVID-19 global pandemic (16, 40). There may be two reasons for this. The first reason may be that relatively stable protective habits were developed after the epidemic and the other was likely the more significant effect of protective attitude and self-efficacy on health behaviors when the epidemic was under control in China. Previous studies indicated that multiple factors affected the eHealth literacy. Shi et al. divided the influencing factors into individual-level factors (age, gender, education, economy, frequency of Internet use, and trust in online health resources), interpersonal-level factors (marital status, family caregivers, and the experience of studying looking for health information), and social/community level factors (language and cultural barriers) based on socio-ecological model with the systematic review methods (41). Levin-Zamir et al. indicated that eHealth literacy was affected by the complexity of the network system (42). When the accessibility and usability of electronic resources were good, that is, the complexity of the network system decreased, the public eHealth literacy would be greatly positively affected. Thus, the comprehensive factors should be considered to improve the level of eHealth literacy from multiple perspectives. Besides, although the eHealth literacy reported by college students was positive in this study, the ability of actual information application may not be optimistic. Kim et al. found that people with a greater ability to seek out information and make judgments had a lower accuracy rate when answering actual questions in Korea (43). This indicated a gap between the subjective self-reporting and objective application results. Neter et al. confirmed a weak correlation (*r* = 0.34) between subjective and objective eHealth literacy among Israeli adults, and proposed that different evaluation tools should be used for evaluation independently (44).

### Study Limitations

This study may have the following limitations: first, data were drawn from one comprehensive university of China and thus the generalizability of this study was limited. Therefore, further multi-center or nationwide investigations were recommended to generalize the findings. Second, the data quality with the online questionnaire may have declined, as the insufficient investigators limited possible oversight throughout when the participants completed the questionnaires. The completion time was used to control the data quality. In addition, this study failed to include college students with lower than a bachelor's degree and broader education levels should have been included. Finally, self-reported eHEALS was used to assess the level of eHealth literacy in this study and it might differ from the objective levels to some extent. It is recommended to further explore the associations between subjective and objective eHealth literacy.

## Conclusions

The results observed that the older female college students, those in good health condition, perceived to have little chance of being infected with COVID-19, and never suspected to suffer from the post-vaccination reaction were more likely to perform better in protective behaviors. This study confirmed the associations of COVID-19 risk perception, eHealth literacy, and protective behaviors. College students with higher eHealth literacy were more likely to engage in positive protective behaviors. Different levels of risk perception significantly predicted the protective behaviors. The dread risk perception and severe risk perception had positive effects in contrast with unknown risk perception which played a negative role. The results suggested that further attention should be given to male, young, and college students in poor health to conduct targeted educational measures following vaccination. Additionally, recommendations were provided by this study for COVID-19 risk management to minimize the negative effects and placing importance to eHealth literacy to support the effective infection control work post vaccination.

## Data Availability Statement

The original contributions presented in the study are included in the article/Supplementary Material, further inquiries can be directed to the corresponding authors.

## Ethics Statement

This study was approved by the Ethics Committee of the Third Xiangya Hospital (ID: I 21071). Online informed consent was obtained from the individuals for the publication of any potentially identifiable images or data included in this article.

## Author Contributions

NQ conceptualized the study, performed the statistical analysis, and drafted the original manuscript. SS, GM, XL, YD, and ZS designed the instrument, collected the data, and participated in revision of the paper. AL and ZZ participated in the design of the study, supervised all the process, and controlled the quality of this study. All authors read and approved the final manuscript.

## Conflict of Interest

The authors declare that the research was conducted in the absence of any commercial or financial relationships that could be construed as a potential conflict of interest.

## Publisher's Note

All claims expressed in this article are solely those of the authors and do not necessarily represent those of their affiliated organizations, or those of the publisher, the editors and the reviewers. Any product that may be evaluated in this article, or claim that may be made by its manufacturer, is not guaranteed or endorsed by the publisher.

## Supplementary Material

Multimedia Appendix 1 The instrument used in the survey.

## Abbreviations

WHO: World Health Organization
CSM-SR: the Common-Sense Model of Self-Regulation
IPC: infection prevention and control
PHERPS: the public health emergency risk perception scale
eHEALS: the eHealth Literacy Scale
VIF: Variance inflation factor.
