# Supplementary material for: Associations of COVID-19 Risk Perception, eHealth Literacy, and Protective Behaviors Among Chinese College Students Following Vaccination: A Cross-Sectional Study
Source: Front Public Health. 2022 Feb 3;9:776829. doi: 10.3389/fpubh.2021.776829 (PMC8850378; doi:10.3389/fpubh.2021.776829)
Supplement: Multimedia Appendix 1 — The instrument used in the survey. [file Data_Sheet_1.docx]

Dear students,

eHealth literacy has played an important role in health information dissemination and disease prevention since the outbreak of Covid-19. Therefore, this study aims to investigate the protective behaviors of Chinese students following vaccinated and to explore the associations of COVID-19 risk perception, eHealth literacy and protective behaviors. It will take 4~6 minutes to complete these questionnaires. Your responses would be of great value to our research. Thank you so much for your cooperation!

2021. 06.09

Do you give consent to participating in this study?

Yes

No

**Part one: Demographic information**

1. Gender

A. Male B. Female

2. Age: years

3.What do you major in?

A. medical subjects

B. non-medical subjects

4.What’s your identity?

A. Student B. Teacher

4.1 what’s your education level?

A. undergraduate

B. post-graduate

C. Ph.D candidate

5. How was your health condition?

A. Very good

B. Pretty good

C. In General level

D. Pretty poor

E. Very poor

6. How was the possibility do you think you might be infected with Covid-19?

A. Impossible

B. Not likely

C. Likely

D. Most likely

7. Have you ever suspected to suffer from post-vaccination reactions following the COVID-19 vaccination?

| ○A. yes | ○B. no |
| --- | --- |

**Part2 COVID-19 Risk perception** **(1~strongly disagree, 2~disagree, 3~not sure, 4~agree, 5~strongly agree).**

|  | Strongly disagree | Disagree | Not sure | Agree | Strongly agree |
| --- | --- | --- | --- | --- | --- |
| 1. The pandemic is highly contagious. | ○ | ○ | ○ | ○ | ○ |
| 2. The pandemic is widespread. | ○ | ○ | ○ | ○ | ○ |
| 3. The health damage caused by the pandemic is fatal. | ○ | ○ | ○ | ○ | ○ |
| 4. I am afraid of being infected. | ○ | ○ | ○ | ○ | ○ |
| 5. I am afraid the people I care about will be infected. | ○ | ○ | ○ | ○ | ○ |
| 6. The pandemic is terrible. | ○ | ○ | ○ | ○ | ○ |
| 7. Not enough is known about the pandemic. | ○ | ○ | ○ | ○ | ○ |
| 8. It is difficult to predict whether a person is infected or not. | ○ | ○ | ○ | ○ | ○ |
| 9. Infections that have occurred may not be accurately detected. | ○ | ○ | ○ | ○ | ○ |

**Part3 eHealth literacy. (1~strongly disagree, 2~disagree, 3~not sure, 4~agree, 5~strongly agree).**

|  | Strongly disagree | Disagree | Not sure | Agree | Strongly agree |
| --- | --- | --- | --- | --- | --- |
| 1. I know how to find helpful health resources on the Internet | ○ | ○ | ○ | ○ | ○ |
| 2.I know how to use the Internet to answer my health questions | ○ | ○ | ○ | ○ | ○ |
| 3. I know what health resources are available on the Internet | ○ | ○ | ○ | ○ | ○ |
| 4. I know where to find helpful health resources on the Internet | ○ | ○ | ○ | ○ | ○ |
| 5. I know how to use the health information I find on the Internet to help me | ○ | ○ | ○ | ○ | ○ |
| 6. I have the skills I need to evaluate the health resources I find on the Internet | ○ | ○ | ○ | ○ | ○ |
| 7. I can tell high quality from low quality health resources on the Internet | ○ | ○ | ○ | ○ | ○ |
| 8. I feel confident in using information from the Internet to make health decisions | ○ | ○ | ○ | ○ | ○ |

**Part4 Protective behaviors following vaccinated. (1~Never, 2~Seldom, 3~Sometimes, 4~Often, 5~Always).**

|  | Never | Seldom | Sometimes | Often | Always |
| --- | --- | --- | --- | --- | --- |
| 1. Keep at least 1 meter away from others in social situations | ○ | ○ | ○ | ○ | ○ |
| 2. Do not wear a mask when going out | ○ | ○ | ○ | ○ | ○ |
| 3. Wash hands promptly after returning to your residence | ○ | ○ | ○ | ○ | ○ |
| 4. Cover any cough or sneeze in your bent elbow |  |  |  |  |  |
| 5. Avoid going to the crowded | ○ | ○ | ○ | ○ | ○ |
| 6. Open windows for ventilation to maintain air circulation | ○ | ○ | ○ | ○ | ○ |
| 7. Cancel non-work or work-related trips | ○ | ○ | ○ | ○ | ○ |

**Thank you for your cooperation~**
